# CD28 is Not Required for T cell Activation

**Published:** 2026

**Authors:** Gongyi Zhang

**Affiliations:** 1 Department of Immunology and Genomic Medicine, National Jewish Health, Denver, USA; 2 Department of Immunology and Microbiology, School of Medicine, Anschutz Medical Campus, University of Colorado, Aurora, USA

**Keywords:** CD28, Anti-CD28, CD3, Anti-CD3, LCK, CD45, PI3K, Galectin

## Abstract

It has been almost four decades since CD28 was identified as a major player (co-stimulator) of T cell activation and has been recognized as one of the major milestones in the T cell field. However, the exact underlying mechanism involved in the activation of CD28 still remains poorly understood. Most importantly, the depletion of CD28 in mice did not lead to a major defect in T cell activation response, even when combined with the deletion of ICOS, a homolog of CD28. Here, we propose that CD28 is just a survival receptor, which works together with CD45 to maintain T cell survival. It does not participate in the activation of the T-cell activation process by antigen-presenting cells (APCs) *in vivo*. Furthermore, we propose that CD28 acts like CD4/CD8 to recruit LCK to activate the PI3 kinase pathway.

## Introduction

The anti-CD28 antibody (Mab 9.3) [1] was found to synergistically enhance T cell response when T cells were activated by anti-CD3 antibodies [2,3]. Furthermore, the anti-CD28 antibody combined with either PHA (Phytohemagglutinin) [1] or PMA (Phorbol Ester) [4,5] could bypass anti-CD3 to activate T cells. A series of research about the function of CD28 led to the assigned functional role of CD28 as the second major signal stimulator for T cell activation in the immunology field. However, the exact underlying mechanism by which CD28 promotes T cell activity remains unresolved, which causes a lot of confusion about how to design effective chimeric receptor-containing CAR-T cells to battle leukemia and other cancers.

## The Underlying Mechanism of Artificial T cell Activation *In Vitro*

A groundbreaking discovery by two groups showed that the addition of an anti-CD3 antibody and an anti-CD28 antibody would trigger the activation and proliferation of T cells *in vitro* [2,3]. Early on, people found that plant lectin proteins were also able to activate T cells [6,7]. The exact underlying mechanism(s) of T cell activation by these artificial proteins remains mysterious. We recently proposed a novel T cell activation model to interpret how these artificial proteins work *in vitro*, which brings the Src kinase family (SKF) member LCK (through CD28) into proximity with the intracellular portions of the TCR; the latter is phosphorylated by LCK to trigger the downstream signal cascade (Zhang, unpublished). At the same time, in that model, we also proposed that CD28 is not required for T cell activation *in vivo* since CD4 and CD8 are capable of recruiting LCK to the proximity of the intracellular portions of TCR subunits. However, we did not provide strong evidence to show why CD28 is not required for T cell activation *in vivo*. Here, we are trying to find evidence to prove this hypothesis.

## T cell *In Vitro* Activation without CD28

In our T cell activation model, we reason that CD4 and CD8 have the ability to replace CD28 to recruit LCK to activate T cells *in vivo* (Zhang, unpublished). This suggests that an anti-CD4 antibody or an anti-CD8 antibody could replace an anti-CD28 antibody to activate CD4 T cells or CD8 T cells *in vitro*, respectively. To our surprise, there is an early report showing that the addition of anti-CD4 antibody and anti-CD3 antibody did trigger the activation and proliferation of CD4 T cells, and it was also true for CD8 T cells, though anti-CD4 antibody alone led to the elimination of CD4 T cells [8]. In this regard, the anti-CD4 antibody and the anti-CD8 antibody could bypass CD28 to activate T cells *in vitro*.

## CD28 is Not Required for T cell Activation *In Vivo*

Based on our current understanding of the conventional TCR activation pathway, APCs (antigen-presenting cells) will present antigens to T cells through their MHC II (MHC I) molecules with the help of CD4 (CD8) to activate the TCR pathway. At the same time, APCs will also contribute CD28 ligands such as CD80 and CD86 to activate CD28. The complete activation of T cells needs the two individual pathways [9]. It was reported that there is a PI3K-binding signature motif (YXXM) within the C-terminus of CD28 [9]. It is also reported that there is an LCK-binding motif at the tip of the C-terminus of CD28 [9], suggesting that LCK could be recruited by activated CD28 and phosphorylate the YXXM motif to recruit PI3K. Interestingly, the LAT (Linker for activation of T cells) adaptor protein not only recruits PI3K but also a large group of other adaptor proteins and enzymes [10]. It suggests that there are other candidates to act as CD28 to recruit essential components for a regular T cell activation process. Furthermore, it was reported that with the knockout of CD28 in mice, T cells still could be activated, though other defects did appear, such as the general survival of T cells without CD28 [11]. Interestingly, from the same report, cytotoxic T cells function normally without CD28 [11]. Another report showed that deletion of CD28 did affect the activation and proliferation of T cells but was just slightly compromised [12]. More impressively, even T cells with a double knockout of CD28 and ICOS were still properly primed, though showing decreased proliferation and reduced IL-4 and IL-10 production as well as compromised antibody class-switching when challenged by pathogens [13]. Surprisingly, a group of memory T cells lost their expression of CD28 on their surface but did not affect their re-activation later [14]. More and more reports showed that there exist T cell populations without CD28 [15]. These results suggest that CD28 may not be required for T cell activation *in vivo*.

## The Exact Function of CD28

LCK, which associates with CD4 and CD8 in T cells, is one of the earliest characterized tyrosine kinases among eight SFK tyrosine kinase family members [16]. Knockout of LCK led to the complete disappearance of T cells [17,18], suggesting it is also essential for T cells to survive, besides the well-known role required for T cell activation (Zhang, unpublished) [19]. It is reported that LCK is constitutively active within T cells [20], which is licensed by CD45 rough removing the phosphor-group on Tyr505 [21] put on by CSK kinase [22–24] (Figure 1), while ZAP70 is only active when T cells are activated [25], supporting the previous discovery that LCK not only is required for T cell activation but also essential for T cells to survive at inactive status. CD45 is expressed on all nucleated hematopoietic cells [26]. Knockout of CD45 in mice led to almost complete loss of mature T cells [27], a similar phenotype to that of double knockout of Lck and Fyn [17] or Lck null alone (a few T cells could survive through a redundant role of Fyn) [18]. Interestingly, LCK is inactive in T cells with CD45 knockout (a few T cells could survive through a redundant phosphatase, CD148) [28], suggesting that CD45 is essential to keep LCK constitutively active. Based on the above analysis, it is likely that CD28 constantly recruits LCK (or is constitutively associated with LCK), which is activated by CD45 *via* removing the phosphate group on Tyr505 (Figure 1). Active LCK then further phosphorylates CD28 to recruit PI3K, which is essential for mature T cells to survive during the inactive status (Figure 1A). A question here is how LCK is brought into proximity to CD45 *in vivo*. One possible scenario is that a rich glycan-binding protein family in the microenvironment (blood circulation), the Galectin protein family, may bring both CD45 and CD28 together (Figure 1A). It was well established that Galectin proteins highly associate with CD45 [29–31], while the direct binding between Galectin proteins and CD28 or other family members started to emerge [32,33].

## The Underlying Mechanism of CAR-T cells

Based on the understanding of T cell signaling, Weiss’ group pioneered the design of chimeric antigen receptors and introduced them into T cells (CAR-T) to treat cancer, first containing only CD3ζ [34], then adding the ITAM motif of CD28 or 4–1BB [35], which were approved by the FDA [36]. An interesting thing is that people do not know why the chimeric receptor with both ITAMs of CD3ζ and CD28 is not only much easier to grow (proliferation) but also more potent than that of both ITAMs of CD3ζ and 4–1BB. One possible interpretation is that ITAMs of CD28 constantly bring LCK (constitutively associated with LCK), which not only activates the PI3K pathway but also phosphorylates CD3ζ to activate the ZAP70 pathway (the regular T cell activation pathway). In this regard, this unique construction of the chimeric receptor (with CD3ζ and CD28) could keep the engineered T cells constitutively active with the help of CD45 (Figure 1B). However, for any differentiated cells, there is a Hayflick limit for proliferation (~50 limited divisions). To increase the efficacy or longevity of these CAR-T cells, there is a need to convert them into memory-like T cells, which have no limitation on cell division.

## Conclusion

Based on the above analysis, it should be safe to claim that CD28 is not required for T-cell activation when T cells are activated by antigen-presenting cells via the TCR-MHC I/II pathways. Instead, CD28 and CD45 work together to keep T cells surviving in normal conditions through the LCK recruitment ability of CD28 *in vivo*, which is constitutively associated with inactive pTyr505 LCK, and could be activated by CD45 (via removing the phosphate group on Tyr505) when they are brought together by Galectin proteins in the circulation.

## Figures and Tables

**Figure. 1.**
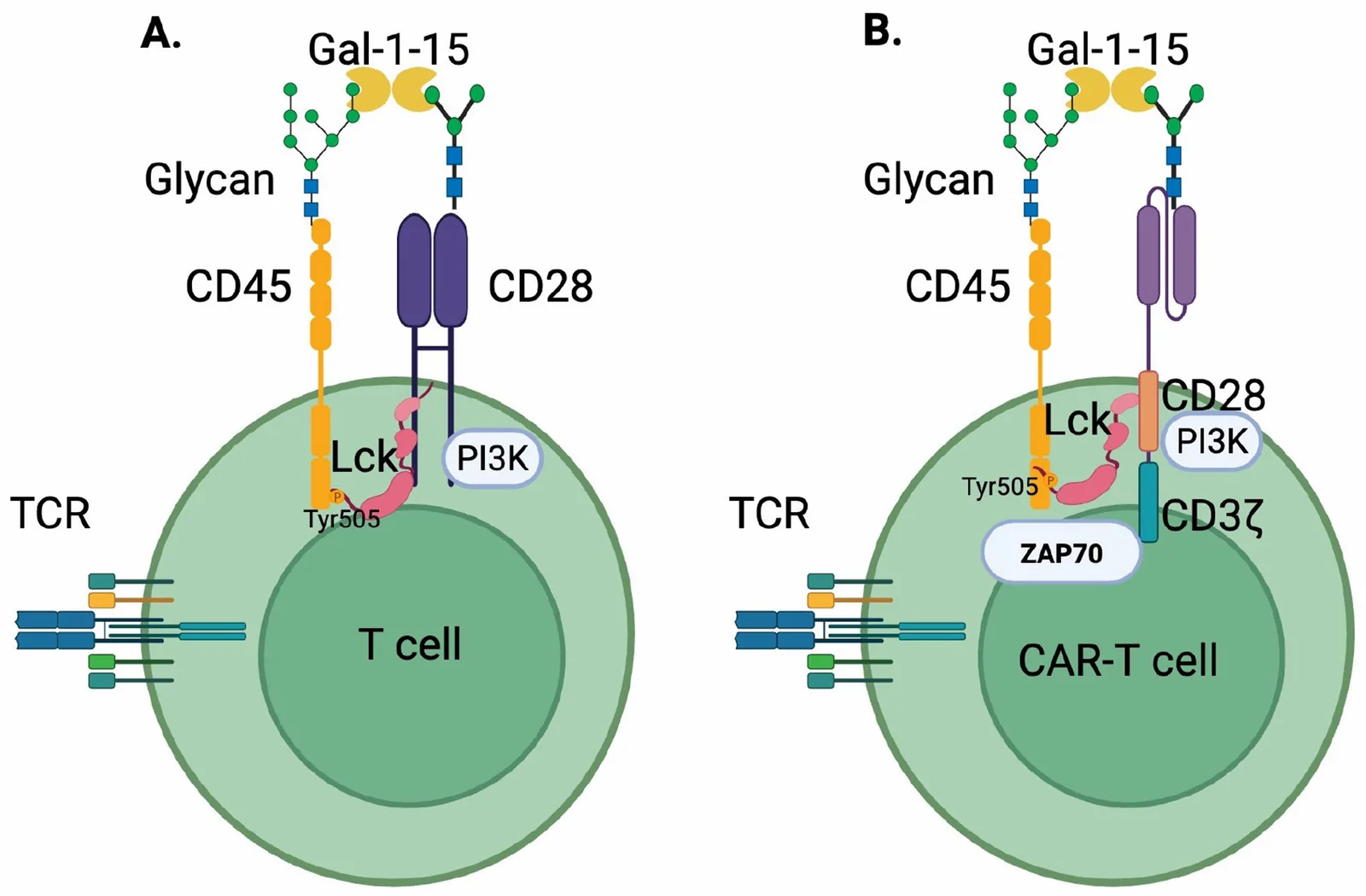
Comparison of a native T cell and a CAR-T cell. **A. Native T cell:** Constitutive signaling through Lck and PI3K is supported by CD45, while galectins bring CD45 and CD28 into proximity. **B. CAR-T cell:** The CAR contains ITAMs associated with CD28 and CD3ζ, which can promote constitutive signaling and proliferation, but may be limited by the Hayflick limit.
